# Lymphatic Valves and Lymph Flow in Cancer-Related Lymphedema

**DOI:** 10.3390/cancers12082297

**Published:** 2020-08-15

**Authors:** Drishya Iyer, Melanie Jannaway, Ying Yang, Joshua P. Scallan

**Affiliations:** Department of Molecular Pharmacology and Physiology, University of South Florida, Tampa, FL 33612, USA; drishya@usf.edu (D.I.); mjannaway@usf.edu (M.J.); yingyang@usf.edu (Y.Y.)

**Keywords:** shear stress, mechanotransduction, cancer, congenital, VE-cadherin, fibrosis, inflammation

## Abstract

Lymphedema is a complex disease caused by the accumulation of fluid in the tissues resulting from a dysfunctional or damaged lymphatic vasculature. In developed countries, lymphedema most commonly occurs as a result of cancer treatment. Initially, impaired lymph flow causes edema, but over time this results in inflammation, fibrotic and fatty tissue deposition, limited mobility, and bacterial infections that can lead to sepsis. While chronically impaired lymph flow is generally believed to be the instigating factor, little is known about what pathophysiological changes occur in the lymphatic vessels to inhibit lymph flow. Lymphatic vessels not only regulate lymph flow through a variety of physiologic mechanisms, but also respond to lymph flow itself. One of the fascinating ways that lymphatic vessels respond to flow is by growing bicuspid valves that close to prevent the backward movement of lymph. However, lymphatic valves have not been investigated in cancer-related lymphedema patients, even though the mutations that cause congenital lymphedema regulate genes involved in valve development. Here, we review current knowledge of the regulation of lymphatic function and development by lymph flow, including newly identified genetic regulators of lymphatic valves, and provide evidence for lymphatic valve involvement in cancer-related lymphedema.

## 1. Introduction

Insult to the lymphatic vasculature, be it through trauma, surgery, or infectious disease, is required for the development of secondary lymphedema. However, the gradual and unclear pathogenesis of cancer-related lymphedema (CRL) argues that the initial damage to the lymphatic vasculature is, by itself, not sufficient to cause overt swelling and fibrosis. Secondary lymphedema does not afflict all cancer patients, indicating that CRL patients may be predisposed to its development or that multiple hits are required to cause CRL as opposed to congenital lymphedema that is caused by single gene mutations. Curiously, secondary lymphedema does not occur immediately after cancer surgery, often taking up to several years to manifest, suggesting that there are likely several pathological processes that promote its development. Most agree that the first step in the development of secondary lymphedema is the impairment of lymph flow.

Over the past decade, many studies have revealed how lymphatic vessels both regulate and are regulated by lymph flow. Two classifications of lymphatic vessels regulate lymph flow using different mechanisms that are relevant to understanding lymphedema [1]. The first type, called lymphatic capillaries, are small blind-ended sacs composed of endothelial cells that begin in the tissues and absorb interstitial fluid that is generated by blood capillary filtration, a vital process that supplies all the tissues and organs with nutrients and oxygen. Once interstitial fluid is absorbed into the lymphatic capillaries, this fluid becomes lymph. However, the pressure in the tissues approaches zero and is not enough to force lymph along the entire lymphatic vasculature. Therefore, the second type of lymphatic vessels, called collecting lymphatic vessels, employ various physiological mechanisms to propel lymph through the lymphatic vasculature to overcome ever-increasing pressures. Collecting lymphatic vessels are larger diameter vessels that are composed of an endothelial layer and a specialized layer of smooth muscle cells that rapidly and forcefully contract to pump lymph along the lymphatic vasculature, similar to the contractions of cardiac myocytes. In addition, the lymphatic endothelial cells (LECs) of collecting lymphatic vessels form specialized bileaflet valves that cooperate with the pumping to keep lymph moving away from the tissues. In general, lymph flows away from the tissues through the lymphatic vasculature to reach lymph nodes to initiate or suppress immune responses and ends in the thoracic duct, which empties lymph into the bloodstream at the subclavian vein to maintain fluid balance in the tissues. If this process becomes dysfunctional, then the tissues will accumulate fluid and become edematous.

## 2. Lymphatic Dysfunction in Cancer-Related Lymphedema

Cancer-related lymphedema (CRL) is a progressive disease characterized by swelling, fibrosis, inflammation, and recurrent infections of the affected limb or tissues [2,3]. In developed countries, the most common population diagnosed with CRL are breast cancer patients [3]. In the US alone, an estimated 5 million people have secondary lymphedema [4]. Inexplicably, CRL usually appears years after surgical removal of lymph nodes as a treatment for cancer metastasis [2], with some estimates stating that 90% of breast cancer-related lymphedema (BCRL) will occur within 2 years [5], while other BCRL patients may experience an onset decades later [2]. Other reports have estimated that breast cancer patients have a 50% rate of developing BCRL within 20 years post-surgery [6]. The earliest pathological steps leading to lymphedema have just begun to be identified, leading to the development of new pharmacological targets for this large patient population.

However, the pathophysiology of CRL remains poorly understood. The simple “stopcock hypothesis” that assumes surgery or trauma blocks lymph flow and leads to an accumulation of fluid and protein [7,8] is insufficient to explain the temporal variability in CRL onset. Some have suggested dividing the pathogenesis into several stages [9]. The first stage affects only the morphology of the lymphatic vessels and lacks obvious edema, which is subsequently followed by a second stage where tissue swelling and fibrosis occur [9]. The initial changes to the lymphatic vessels include loss of contractile function, lymphatic valve incompetence, and vessel dilation, whereas in chronic lymphedema, the retrograde flow of lymph into the dermis of the skin is observed and collateral lymph flow may be present [9]. Several hypotheses have been proposed by clinicians to explain the pathogenesis of lymphedema along with its delayed onset.

Based on the clinical features of lymphedema, it was recently hypothesized that surgery causes a reduction in lymph flow which initiates a feed forward cycle of inflammation, leading to tissue fibrosis that then inhibits lymphatic function and further reduces lymph flow [10]. Using the mouse tail as a model, this landmark study showed that lymphedema was associated with the production of Th2 cytokines. Further, it demonstrated that removal of CD4^+^ T-cells or antibody depletion of the Th2 cytokines, IL4 and IL13, prevented the inflammation, fibrosis, and swelling in the mouse tail lymphedema model. The same approach was able to decrease the swelling that had already developed. Follow-up studies have confirmed the critical role of T-cells and the Th2 inflammatory response in other animal models of lymphedema [11,12,13].

Confirming a crucial role for inflammation, it was recently discovered that the non-steroidal anti-inflammatory drug (NSAID) ketoprofen was able to reverse edema and fibrosis of experimental lymphedema in mice [14]. However, further investigation revealed that the mechanism of action of ketoprofen was via off-target inhibitory effects on 5-lipoxygenase that abrogated the production of leukotriene B_4_ (LTB_4_). At low doses, LTB_4_ was found to increase the expression of the lymphatic growth factor receptor, VEGFR3, whereas high doses similar to those found in chronic lymphedema inhibited both the VEGFR3 and Notch1 pathways [15]. A clinical trial has been completed evaluating ketoprofen as a treatment for lymphedema patients [16]. Patients treated with ketoprofen exhibited reduced skin thickness, better scores of histopathology, and decreased expression of G-CSF. Since ketoprofen was well-tolerated in the clinical trial and is already FDA approved, the results hold promise as a potential treatment for CRL.

Following axillary lymph node removal, it is known that lymph outflow from the arm is reduced [7,17]. Based on this knowledge, it has been hypothesized that the collecting lymphatic vessels fail to pump due to a chronic exposure to elevated afterload, similar to cardiomyocytes during heart failure [7,17,18]. Although collecting lymphatic vessels can adapt to increases in afterload by increasing their contractility, there is a limit to how much pressure the collecting lymphatic vessels can pump against [19]. A point is reached at which the lymphatic drainage rate falls below the blood capillary filtration rate, which ultimately manifests as the swelling characteristic of CRL. Using lymphatic congestion lymphoscintigraphy, the pumping ability of collecting lymphatic vessels was found to be lower in BCRL and was proportional to the amount of swelling, suggesting pump failure as a potential cause of edema [18]. Later studies were performed to identify factors that predicted which patients will eventually develop BCRL. Patients predisposed to developing BCRL had higher lymphatic vessel pumping pressures prior to surgery [20], which is puzzling because this indicates that the collecting lymphatic vessels in these patients can handle higher pressure loads (e.g., afterload). The same study found that post-surgery, the patients that developed BCRL had dramatically decreased interstitial tracer clearance by the arm lymphatic vasculature, suggesting an increased afterload or an increased outflow resistance due to vessel remodeling [20], both of which would be expected to impair lymphatic pumping. Impaired lymphatic pumping was recently demonstrated to be sufficient to severely inhibit lymph flow in vivo in mice, but only when a gravitational load was applied to mimic human limbs [21]. However, it remains unknown whether impaired lymphatic pumping alone is sufficient to induce lymphedema in humans.

While these hypotheses are based on gradual, progressive processes that offer explanations for why BCRL does not develop immediately post-surgery, there are still several questions that remain. For example, how does reduced lymph flow initiate inflammation? Is lymphatic endothelium also involved in the pathogenesis of lymphedema? Are lymphatic valves involved in CRL pathogenesis? In the next sections, we will outline and propose a possible answer to these questions.

## 3. Lymph Flow, Lymphatic Function, and Lymphatic Valves

The lymphatics play an important role in maintaining tissue fluid homeostasis by absorbing interstitial fluid in the form of lymph and returning it into the central circulation. Lymph formation involves movement of fluid from interstitial tissues into lymphatic capillaries and this requires a downhill pressure gradient between these compartments that is established by periodic external forces such as cardiac contractions, arterial pulsation, respiration, peristalsis of the gut, and skeletal muscle contractions (e.g., the skeletal muscle pump of the lower leg in humans). However, these external forces are not sufficient to transport lymph against a pressure gradient to the bloodstream in larger mammals, such as humans, where the force of gravity directly opposes the vertical transport of lymph.

The smooth muscle cells covering collecting lymphatic vessels, therefore, have an inherent contractile activity that enables them to actively regulate lymph flow. Lymphatic muscle cells circumferentially wrap collecting lymphatic vessels and exhibit regular, periodic, and forceful contractions that generate the pressure required to move lymph flow along the lymphatic vessel [21,22]. Regularly spaced bileaflet valves shorten the distance that lymph needs to move, helping to reduce the amount of pressure that needs to be generated locally by pumping [22]. Together, lymphatic pumping and the intraluminal valves cooperate to enable the efficient movement of lymph against a progressively increasing pressure before it is emptied into the great veins that have pressures of 10–16 cmH_2_O in humans. Due to the combination of lymphatic pump activity and external forces, the collecting lymphatic vessels experience constant oscillations in pressure and flow [22,23]. Additionally, the lymphatic vasculature does not form a full circulatory system, so the flow velocity of lymph is slower relative to the blood vasculature [24].

Lymph flow exerts a physical frictional force on the lymphatic endothelium called shear stress. Shear stress experienced by LECs lining the vessel can vary in magnitude and direction depending on local lymph flow dynamics. A study of mesenteric collecting lymphatic vessels in vivo described lymph flow as phasic with lymph flow velocity and shear stress being determined by the contraction cycle [24]. While the average shear stress was calculated to be ~0.7 dynes/cm^2^, this is a poor indicator of the maximum shear stress that the endothelium experiences because lymph flow is oscillatory. Thus, the peak shear stress was between 2.5 and 12 dynes/cm^2^ [24]. Brief periods of flow reversal resulted in lymph flow that was approximately 90% in the orthograde direction and 10% in the retrograde direction [24]. While this study was performed in the adult rat, the oscillatory nature of lymph flow has been confirmed in the mouse [25], which exhibits spontaneous contractions [26] and is a widely used model to investigate lymphatic biology and disease.

The physiological effect of shear stress on lymphatic function was demonstrated in a study wherein collecting lymphatic vessels were surgically removed from the mesentery and tied onto glass cannulas that controlled the pressure and flow through the vessel [27]. Using this elegant approach, it was demonstrated that shear stress potently inhibited lymphatic pumping through the production of nitric oxide (NO) [27]. More recently, it has been shown that while any amount of NO inhibits lymphatic vessel pumping, high doses can completely prevent pumping [26]. Inflammation was found to elicit high levels of NO production by immune cells adjacent to collecting lymphatic vessels to inhibit pumping [28]. Inflammation-induced NO production by T-cells inhibited lymphatic pumping in a mouse model of lymphedema, indicating that NO likely inhibits lymphatic pumping in human CRL patients [29].

Other studies have shown that lymphatic endothelium is highly sensitive to the direction and magnitude of lymph flow. In a mouse model of vascular malformation, redirection of blood flow through the lymphatic vasculature resulted in LECs losing the expression of the transcription factor responsible for conferring lymphatic fate, *Prox1* [30]. Additionally, exposure to laminar flow altered the signaling of lymphatic endothelium to induce sprouting lymphangiogenesis [31]. Together, these studies indicate that lymphatic vessels are adapted to a low magnitude oscillatory flow. Interestingly, this type of flow and shear stress is similar to that which causes atherosclerosis in disturbed flow regions of large arteries [32].

One of the most intensely studied roles of shear stress in the lymphatic vasculature has been the discovery that oscillatory shear stress drives the formation of lymphatic valves, which are crucial for maintaining forward lymph flow [25,33]. Valve development begins during embryogenesis when a subset of lymphatic endothelial cells upregulates the transcription factors *Prox1*, *Foxc2* and *Gata2* to become specified to form valves [25,34]. After specification, these valve endothelial cells undergo stepwise morphological changes and migrate to give rise to fully formed bileaflet valves [25]. Implicating lymph flow as a major contributor to valve formation, it was shown that valve formation began immediately after the onset of lymph flow during embryogenesis [25]. This was followed by a study demonstrating that the lack of lymph flow in vivo resulted in a failure to form lymphatic valves [33]. Most recently, we found that a key regulator of mechanotransduction signaling is required for lymphatic valve formation [35]. Therefore, shear stress generated by lymph flow is a major driver of lymphatic valve formation.

After their formation, lymphatic valve leaflets require constant lymph flow and shear stress signaling throughout life to escape cell death [34,35,36]. Thus, lymphatic valve leaflets are not permanent structures and will disintegrate in the absence of lymph flow through apoptosis. The concept of valve maintenance is especially relevant to CRL patients, where lymph flow after lymph node removal surgery is significantly reduced or absent.

Physiologically, lymphatic valves open and close passively in response to pressure gradients exerted across the valve leaflets [37]. If the downstream pressure is higher than the upstream pressure, the valve closes to prevent backwards flow, and if the upstream pressure is higher, then the valve leaflets open to permit forward flow. However, a minimum pressure gradient is required to close the valves, which is dependent on the vessel diameter, and this explains why a small amount of backwards flow can occur immediately prior to valve closure. If pressures are equal on both sides of the valve, the leaflets are biased to be in the open position [37]. Valves also serve to divide the total pressure gradient required for forward lymph flow into a series of small pressure gradients. For instance, when the force of gravity is considered in an upright human, it becomes apparent that a column of fluid in the legs, uninterrupted by valves, would have a very high hydrostatic pressure due to its location below heart level (approximately ≥60 cmH_2_O). Therefore, the small pressures generated by pumping alone are insufficient to push lymph upwards through the legs, requiring lymphatic valves that prevent retrograde lymph flow and break up the pressure gradients to allow forward lymph flow. The same is true for the upper limbs, most of which hang below the level of the heart. Notably, BCRL patients have swelling of the upper limbs, while congenital lymphedema patients may have swelling in the lower and/or upper limbs. Over the past two decades, much has been discovered about the genetic regulators of lymphatic valve development, which will be discussed next.

## 4. Genetic Models of Congenital Lymphedema

The identification of gene mutations in patients presenting with congenital lymphedema has fueled the development of mouse models to gain insight into the genetic mechanisms underlying the development of lymphedema. Many of these studies have identified roles for these genes not only in lymphangiogenesis but also in valve formation and postnatal valve maintenance, thus supporting the assertion that valve defects likely underlie the initiation of congenital lymphedema and potentially CRL. Here, we review some of the genes that have been identified in congenital lymphedema patients and discuss their corresponding mouse models.

The VEGFC/VEGFR3 signaling pathway is the central lymphangiogenic pathway that promotes lymphatic vessel growth from preexisting lymphatic vessels. During embryonic development, a subpopulation of blood endothelial cells in the cardinal vein begin to express the fate-determining transcription factor PROX1 to acquire a lymphatic identity. Due to a high concentration of VEGFC in the extracellular matrix outside the cardinal vein, these PROX1-positive cells begin to migrate and proliferate to form the lymphatic vasculature. This process is mediated by intracellular signaling events initiated upon binding of VEGFC to the tyrosine kinase receptor, VEGFR3, on the surface of LECs. Congenital mutations in *FLT4*, which encodes VEGFR3, have been linked to Milroy disease which is characterized by the onset of lymphedema in the lower limbs. Heterozygous missense mutations in the tyrosine kinase domain of the gene prevent the autophosphorylation of VEGFR3 which impairs its downstream signaling and affects lymphatic vessel development [38,39]. A similar mutation was discovered in the *Chy* mouse strain that has impaired VEGFR3 signaling and is characterized by dermal lymphatic hypoplasia, lower limb swelling, and chylous ascites [40]. These studies have concluded that impaired lymphangiogenesis in humans and mice with this mutation affects uptake of interstitial fluid and leads to the observed lymphedema phenotype. However, it is currently unknown whether VEGFR3 plays a role in other aspects of lymphatic growth, such as valve development, and how that may contribute to the phenotype in Milroy patients. Additionally, VEGFR3 appears to be a target of LTB_4_ signaling in CRL patients [15], which argues that VEGFR3 has other roles in lymphatic function besides regulating development and lymphangiogenesis.

The PI3K/Akt/mTOR pathway acts downstream of VEGFR3 activation to promote lymphangiogenesis [41,42]. Homozygous deletion of *Pik3r1* and *Pik3ca*, which encode the regulatory and catalytic subunit of PI3K, respectively, caused defects in lymphatic sprouting and led to chylous ascites [43,44,45]. These studies also identified a role for PI3K in lymphatic vascular remodeling; mesenteries of *Pik3r1* null mice failed to remodel into a mature lymphatic network and lacked lymphatic valves [44]. AKT is one of the major downstream targets of PI3K and *Akt1*^−/−^ mice exhibited impaired LEC proliferation and remodeling defects as evidenced by reduced lymphatic capillary diameter, lack of lymphatic valves, and sparse smooth muscle coverage [46]. In the context of human disease, somatic activating mutations in components of the PI3K/AKT pathway have been identified in overgrowth syndromes such as Proteus, CLOVES (congenital lipomatous overgrowth, vascular malformations, epidermal nevi, and skeletal/spinal abnormalities) and Klippel–Trenaunay–Weber syndromes. Patients with these syndromes exhibit lymphatic malformations (LMs), which are localized lesions comprised of dilated blood-filled lymphatic vessels that are disconnected from the lymphatic network. Further studies using mouse models with gain-of-function mutations in the PI3K/AKT pathway are needed to understand the etiopathology of LMs and role of PI3K/AKT/mTOR in lymphatic function.

Lymphedema distichiasis (LD) is an autosomal dominant disorder that is characterized by variable onset lymphedema in the lower limbs and the presence of an extra row of eyelashes (i.e., distichiasis) [47]. Identification of gene mutations in *FOXC2* in patients with LD led to the development of *Foxc2^+/−^* mice to study the pathogenesis of LD [48]. Because the *FOXC2* gene contains a single exon, most mutations lead to a loss of the protein product and therefore global heterozygous mice are an accurate model for mutations in this gene [48,49]. Lymphangiography studies performed on LD patients revealed lymphatic vessel hyperplasia, ectopic pericyte recruitment to lymphatic capillaries, and retrograde lymph flow, all of which were phenocopied in *Foxc2* mice [48,49,50]. FOXC2 was later discovered to be a transcription factor required for the formation of lymphatic valves in mice [49]. Interestingly, when both alleles of the *Foxc2* gene were conditionally deleted from the lymphatic vasculature in postnatal mice, this led to a complete loss of lymphatic valves after many had already formed [36]. This led to the concept that lymphatic valves not only require lymph flow signals for their formation, but also require flow signaling throughout life to maintain the valve leaflets. Therefore, these findings suggest that lymphedema in lymphedema distichiasis patients may be initiated by the loss of valves due to haploinsufficiency of *FOXC2*.

Heterozygous germline mutations in *GATA2* resulting in haploinsufficiency of the gene have been shown to underly Emberger syndrome [51,52]. Patients with this syndrome exhibit primary lymphedema and are predisposed to developing myelodysplastic syndrome/acute myeloid leukemia (MDS/AML). Based on these findings, the role of GATA2 was investigated in the murine lymphatic vasculature [34]. GATA2 was highly expressed in the LECs of valve leaflets and in vitro experiments revealed that GATA2 controlled the expression of genes involved in valve development such as *Prox1*, *Foxc2*, *Itga9*, and *Nfatc1* [34]. In a subsequent study, GATA2 was found to regulate valve specification during embryogenesis by directly binding enhancer elements in the *Prox1* and *Foxc2* locus and upregulating their expression in response to oscillatory shear stress [34]. Consistent with these data, lymphatic-specific deletion of GATA2 during embryonic development led to fewer valve specification cell clusters at E16.5 and a complete lack of fully formed valves at E18.5 [34]. Additionally, the mutations in *GATA2* that underlie Emberger syndrome produced mutant GATA2 proteins that were unable to bind enhancer elements in both the *PROX1* and *FOXC2* genes that were active only in the valve cells, and only these mutations were associated with lymphedema in patients. This study concluded that the *GATA2* mutations abolishing enhancer-binding resulted in the loss of valves due to reduced expression of PROX1 and FOXC2, and strongly suggests that a loss of valves leads to lymphedema in human patients [34].

Generalized lymphatic dysplasia (GLD) presents as facial and full body edema along with some cases of pulmonary and intestinal lymphangiectasia, pleural effusion, chylothorax, and pericardial effusion [53]. Hennekam lymphangiectasia–lymphedema syndrome (HS), characterized by severe facial swelling, seizures, and mental retardation, was the first described autosomal recessive GLD [54]. Sequencing studies revealed that mutations in *CCBE1*, *FAT4*, and *ADAMTS3* underlie this syndrome and this has led to the development of mouse models to understand how mutations in these genes cause the lymphatic phenotypes associated with HS [55,56,57,58]. Studies have shown that CCBE1 and ADAMTS3 play critical roles in the VEGFR3/VEGFC signaling pathway in order to promote lymphangiogenesis. CCBE1 is secreted into the extracellular matrix where it enhances the ADAMTS3-mediated proteolytic cleavage of VEGFC into its active form [59]. The active form of VEGFC can then initiate signaling through VEGFR3, which regulates the sprouting and migration of LECs during lymphangiogenesis. In agreement, *Ccbe1*^−/−^ and *Adamts3*^−/−^ embryos exhibited severe edema and were completely devoid of lymphatic vessels due to a failure of PROX1-positive LECs to migrate out from the cardinal vein [60,61]. While there is no evidence for the role of CCBE1 and ADAMTS3 in valve morphogenesis, phenotypic differences between Milroy Syndrome and HS suggest that there may be additional non-lymphangiogenic functions of these genes that are currently unknown. In support of this, recent studies into the function of FAT4 in lymphatic development suggest that valve defects may contribute to the lymphedema phenotype in Hennekam patients. These studies identified a role for FAT4 in maintaining LEC polarity in response to lymph flow, which is important for valve morphogenesis [62,63]. The studies collectively showed that in *Fat4*-deficient embryos, valve endothelial cells were unable to orient along the direction of flow due to impaired cell polarization and this affected cell migration and consequently valve leaflet formation [62,63].

The incidence of a distinct cohort of GLD patients exhibiting mild facial edema, no seizures, and normal intelligence led to whole exome sequencing studies that identified a new category of autosomal recessive GLD that was caused by mutations in the gene *PIEZO1* [64,65]. PIEZO1 is a mechanosensitive cation channel found on endothelial cells that activates intracellular signaling pathways in response to mechanosensory stimuli like fluid shear stress. Identification of loss-of-function mutations in *PIEZO1* in GLD patients prompted the investigation of the role of this ion channel in lymphatic development and function. In mice, it was later discovered that PIEZO1 was required for valve morphogenesis during embryonic development [66]. This study found that endothelial-specific deletion of *Piezo1* caused the mice to develop pleural effusion and die within 2 weeks after birth, which was associated with an impairment in valve leaflet protrusion into the lumen leading to drastically fewer valves [66]. This study proposed that activation of PIEZO1 in response to shear stress from lymph flow stimulates LEC migration, actin polymerization, and cell junction remodeling in order to facilitate valve leaflet formation [66]. A subsequent study confirmed the role of PIEZO1 as a shear sensor that is required not only for valve development during embryogenesis, but also for valve maintenance throughout adult life [67]. These findings support the idea that valve defects caused by specific genetic mutations may underlie lymphedema initiation and progression.

Integrin-α9 is a receptor on LECs that binds fibronectin-EIIIA (FN-EIIIA) that is a component of the extracellular matrix. Mice lacking integrin-α9 develop chylothorax and die of respiratory failure postnatally [68]. In humans, a study identified heterozygous missense mutations in *ITGA9* in fetuses with severe congenital chylothorax prompting further studies into the role of integrin-α9 in lymphatic vessel development [69]. Integrin-α9 was found to be highly expressed in valve endothelial cells and was critical in facilitating valve leaflet elongation through its interaction with FN-EIIIA [70]. The specific role of integrin-α9 in valve development led to the loss of valves in *Itga9^−/−^* mice, which was responsible for leakage of lymph into the thoracic cavity and death of these mice postnatally [70]. More importantly, since no other defects were identified besides the loss of valves, this study further supports the concept that the loss of valves initiates lymph leakage and lymphedema. Additional support for this concept comes from a study in mice demonstrating that loss of valves in the paravertebral lymphatic vessels leads to chylothorax [71].

Capillary malformation-arteriovenous malformation (CM-AVM) is a vascular disorder that is associated with mutations in the gene *RASA1*. A small subset of patients harboring this mutation have also been found to exhibit lymphatic abnormalities such as lymphedema in the upper and lower extremities [72,73], chylous ascites [74], chylothorax [74] and swelling with lymphatic vesicles [73]. Near-infrared fluorescence lymphatic imaging (NIRFLI) and radiographic lymphangiography performed on a CM-AVM patient with a *RASA1* mutation revealed hyperplasia of lymphatic capillaries, abnormally dilated collecting vessels, and disrupted lymph flow [73]. Using inducible lymphatic-specific *Rasa1* knockout mice, RASA1 was initially identified as a negative regulator of LEC proliferation, making it critical for preventing lymphatic vessel overgrowth in adult animals [75]. Although this explained the lymphatic hyperplasia observed in *Rasa1*-deficient mice, it failed to explain the appearance of chylothorax. The same group later found that deletion of *Rasa1* resulted in LEC apoptosis within valve leaflets, resulting in dysfunctional valves that were unable to seal tightly (i.e., prevent backward fluid leakage) against an adverse pressure gradient [76]. The lymphatic valve defects were at least partly responsible for lymph leakage into the thoracic cavity and further support the concept that the loss of valves initiates lymph leakage and chylothorax [76].

In conclusion, a mutation in any one of the many genes reviewed here leads to valve defects in the corresponding genetic mouse model. The repeated finding of valve defects in mouse models of congenital lymphedema mutations strongly supports the idea that in humans with the same mutations, valve defects cause or significantly contribute to the lymphedema (Table 1). Even genes previously identified to cause congenital lymphedema that were not initially reported to exhibit valve defects in the knockout models have recently been demonstrated to harbor valve defects (e.g., RASA1 [75,76]). The presence of valves in animal models should not be assumed to imply the absence of valve defects because mutations can cause a reduction, not a complete loss, in the number of valves and/or reduce the valve leaflet length (e.g., FOXC2 [77]). Given that valve defects are such a common theme in congenital lymphedema, it is logical to suggest that defective valves may also play a significant role in the pathophysiology of CRL. Further, since lymph node surgery causes a loss of lymph flow in CRL, and we know that lymph flow is required for the constant maintenance of valves, there is ample evidence to support the involvement of defective lymphatic valves in CRL. In agreement with this, dermal backflow is used as a diagnostic criterion for CRL and dermal backflow of lymph can only occur in the presence of valve regression or defects. In conclusion, we propose that valve regression not only causes congenital lymphedema, but likely contributes significantly to the pathogenesis of CRL. In the following section, we will discuss new evidence for how lymph flow regulates lymphatic valve development.

## 5. How Lymph Flow Regulates Lymphatic Valve Development

Knockout mouse models have revealed that many genes cooperate to orchestrate valve formation and maintenance, a potential contributor to the pathophysiology of lymphedema. As discussed in the preceding section, many of the genes that are mutated in congenital lymphedema patients are intracellular signaling molecules or transcription factors located in the cell nucleus of LECs. However, this fact highlights a glaring problem—how do intracellular proteins know when shear stress is altered or impaired? Are there proteins on the cell surface that transmit information about shear stress to proteins inside the cell? These questions are particularly relevant because the majority of pharmaceutical targets are comprised of ion channels and receptors on the cell surface.

Recently, mutations in *PIEZO1* were identified in human lymphedema patients, which encodes an ion channel that is sensitive to mechanical force. In the blood vasculature, others have identified a role for PIEZO1 in responding to shear stress and even differentiating between laminar and oscillatory shear stress [83,84]. In the lymphatic vasculature, deletion of the *Piezo1* gene in mice leads to a severe reduction in the number of valves and pleural effusion [66,67]. While it is known that the opening of PIEZO1 leads to calcium influx into the cell, the exact molecular mechanism for how calcium regulates the intracellular signaling and transcriptional changes required for valve development remains unknown.

So, what is the link between lymph flow and changes in gene transcription? To help address this question, we looked to how blood endothelial cells (BEC) respond to blood flow. In 2005, a protein complex was identified at cell-cell junctions that regulated endothelial cell signaling in response to shear stress [85]. This mechanotransduction complex was composed of PECAM1, VE-cadherin, and VEGFR2, and in blood endothelium the loss of either PECAM1 or VE-cadherin abrogated all signaling in response to flow. While *Pecam1*^−/−^ mice were reported to exhibit defective lymphatic valve formation in the embryo, numerous valves remained and these mutant mice survived to adulthood with no apparent defects [86], indicating that mechanotransduction signaling in LECs may not depend as strongly on PECAM1 as it does in BEC. Therefore, we generated a conditional floxed *Cdh5* allele to enable the lymphatic-specific deletion of VE-cadherin to study its role in lymphatic valve development [35].

Embryonic deletion of VE-cadherin led to the complete absence of lymphatic valves, including the lymphovenous valves at the junction of the thoracic duct and subclavian vein [35]. In addition, the nuclei of the LECs failed to align to the direction of flow in the absence of VE-cadherin. To conclusively demonstrate that VE-cadherin was required for flow signaling, we cultured control and VE-cadherin-deficient LECs in the presence and absence of oscillatory shear stress and probed for the expression of valve transcription factors. When VE-cadherin was silenced, LECs failed to upregulate the mechanoresponsive genes FOXC2, GATA2, and KLF4. Collectively, these data strongly indicate a role for VE-cadherin in mechanotransduction signaling in the lymphatic vasculature. To determine whether VE-cadherin regulated valve maintenance after birth, we induced the deletion of VE-cadherin postnatally after many lymphatic valves had already formed and counted the valves 1 and 2 weeks later. Postnatal deletion of VE-cadherin led to a severe regression of lymphatic valves that worsened over time, demonstrating that VE-cadherin signaling is required for lymphatic valve maintenance.

To gain insight into the mechanisms of how VE-cadherin regulated valve development, we focused on the well-known VE-cadherin binding partner, β-catenin, because it had been reported that β-catenin was required for lymphatic valve formation in the embryo by directly binding the promoters of *PROX1* and *FOXC2* [87]. Using a knockdown approach in vitro that was confirmed by immunostaining in vivo, we demonstrated that the expression of β-catenin was completely lost in VE-cadherin-deficient LECs [35]. This finding indicated that VE-cadherin sequesters β-catenin at the cell membrane to prevent its degradation by its destruction complex and in the absence of VE-cadherin, β-catenin is fully degraded. To confirm a role for β-catenin downstream of VE-cadherin, we expressed a mutant form of β-catenin that cannot be targeted by its destruction complex but can still bind VE-cadherin. When this β-catenin mutant was expressed on the VE-cadherin-deficient background, it prevented approximately half of the valve regression. This argued that another signaling pathway existed downstream of VE-cadherin in LECs.

The canonical mechanotransduction signaling complex originally identified in BEC was shown to stimulate PI3K/AKT signaling instead of β-catenin [85]. Therefore, we exposed cultured LECs to oscillatory shear stress and silenced VE-cadherin expression, revealing that the loss of VE-cadherin inhibited AKT phosphorylation [35]. To confirm that AKT signaling was the second pathway that regulated valve development, we administered a chemical activator of AKT to VE-cadherin-deficient mice. Augmenting AKT signaling in LECs led to an approximately 50% rescue in the number of valves, similar to the β-catenin rescue experiment. In conclusion, we identified that VE-cadherin regulated both β-catenin and AKT signaling in response to oscillatory shear stress, and that these mechanotransduction signaling events are required for lymphatic valve formation and maintenance [35].

## 6. Evidence for Valve Defects in Cancer-Related Lymphedema

Although lymphatic valve incompetence is often cited as a characteristic of lymphedema in patients, this is based on indirect evidence using diagnostic imaging techniques that demonstrate retrograde lymph flow as an indicator of valve incompetency. No direct, quantitative evidence of valve defects—such as regression or defective morphology—has been shown in human patients with either congenital or cancer-related lymphedema. This is most likely due to the small size of lymphatic valves and the poor resolution of current clinical imaging modalities. However, lymphangiography has been used to show that in CRL patients, the lymphatic vessels become larger in diameter [88,89], which is thought to lead to valve incompetence [90]. More recently, it has become appreciated that dermal backflow on lymphoscintigraphy is a diagnostic criterion for CRL [2] and the only way that dermal backflow can occur is if lymphatic valves are missing or are defective, failing to seal properly.

Many of the numerous mouse models of congenital gene mutations exhibit lymphatic valve defects, including *Foxc2*, *Gata2*, *Rasa1*, *Piezo1*, *Fat4*, *Itga9*, and *Akt1* (discussed above). While confirmation of valve defects in humans is currently lacking, isotope lymphoscintigraphy in lymphedema patients with mutations in *FOXC2* demonstrate abnormally low tracer uptake and dermal backflow [50], again indicating that these patients likely exhibit valve defects that may initiate the development of lymphedema. Similarly, duplex ultrasound showed that patients with mutations in *FOXC2* have severe venous valve failure as measured by pathological venous reflux in several segments of veins in the lower limbs [91]. Whilst these imaging techniques provide strong evidence for valve incompetence, imaging modalities capable of detecting lymphatic vessel structure, including the visualization of lymphatic valves, are needed to confirm these data.

Studies have identified several gene mutations (e.g., GJC2, FOXC2) in the form of single nucleotide polymorphisms in CRL patients [92,93,94,95,96]. Such gene mutations may represent a genetic predisposition for CRL, offering a potential explanation for why only a subset of patients undergoing surgical intervention eventually develop lymphedema and suggesting that the pathophysiology of CRL could be more similar to congenital lymphedema than previously appreciated [97].

## 7. Conclusions

**Figure 1:**
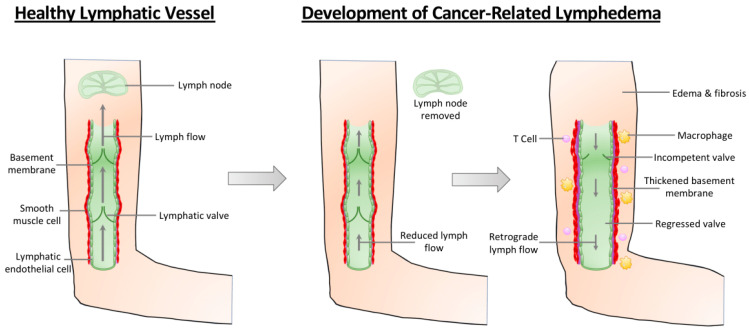
Pathological changes in the arm during the development of cancer-related lymphedema. In a healthy lymphatic vasculature (leftmost panel), valves prevent the retrograde flow of lymph, ensuring its efficient transport from the lymphatic capillaries to the lymph node. Following surgical intervention as part of cancer therapy, some patients will develop cancer-related lymphedema. Lymph node removal surgery reduces the flow of lymph through the lymphatic vasculature (middle panel). Over time, however, collecting lymphatic vessels exhibit increased inflammation, thickened basement membrane, increased smooth muscle cell coverage, and dilation (right panel). Reduced lymph flow results in reduced VE-cadherin-dependent mechanotransduction signaling that reduces the expression of transcription factors required for valve maintenance, leading to valve regression. Consequently, lymph flow moves in the retrograde direction and fluid is no longer transported out of the tissue, resulting in lymphedema and fibrosis.

To summarize, studies have shown that there is a loss of lymph flow after lymph node removal surgery in CRL patients [2,7,17]. Lymph flow and shear stress were demonstrated to be required for valve maintenance by several studies [33,36,66,67]. Further, we have demonstrated that VE-cadherin-dependent mechanotransduction signaling is required for lymphatic valve maintenance by controlling the expression of lymphatic valve transcription factors [35]. In human patients, dermal backflow is used as a diagnostic criterion for lymphedema and the retrograde flow of lymph can only occur if valves are missing or dysfunctional [2]. Finally, there is abundant evidence that lymphatic valves are defective in congenital lymphedema patients supporting a causative role in these patients. Thus, integrating these studies, we propose that reduced VE-cadherin-dependent signaling occurs in response to the loss of lymph flow that occurs after lymph node removal surgery in CRL patients (Figure 1). The loss of VE-cadherin-dependent shear stress signaling, but not necessarily the loss of VE-cadherin expression, will then cause valve regression based on our results with lymphatic-specific deletion of VE-cadherin in mice. The loss of lymphatic valves will then cause a greater reduction in lymph flow and allow dermal backflow to occur. Therefore, lymphatic valves should be evaluated in CRL patients in future clinical studies.

Thus, a goal for future investigation is to directly show whether human CRL patients have lymphatic valve regression or other valve defects. To accomplish this, there would need to be a way to visualize and image the lymphatic valves in vivo. Since near-infrared imaging has been used to map lymph flow in human lymphedema patients, perhaps this technique could be modified to label the valve leaflets without harming the patient. In lieu of this approach, mouse models of CRL should be employed to study whether lymph node removal affects lymphatic valve maintenance in vivo, and whether inflammation plays any role in valve regression. Genetic approaches to rescue valve regression could also be combined with animal models of CRL to implicate valve regression in this poorly understood disease.

Our study demonstrating that constant VE-cadherin expression and signaling are required for the lifelong maintenance of lymphatic valves has potential consequences relevant to CRL [35]. Because previous studies have established a role for Th2 inflammation in initiating lymphedema after lymph node removal [10,11,12,13], and inflammatory cytokines are known to downregulate VE-cadherin expression in LECs [98], a logical consequence is that perhaps inflammation may inhibit mechanotransduction signaling. In support of this idea, a previous study showed that the Th2 cytokine, IL4, disrupts VE-cadherin expression in BEC [99]. If confirmed in the lymphatic vasculature, then this implies that inflammation may negatively regulate mechanotransduction signaling by inhibiting VE-cadherin expression, which would be expected to cause valve regression. This mechanism could also explain the delay in onset of CRL because residual VE-cadherin would likely remain, leading to a slower valve regression than occurs in the knockout animals lacking all VE-cadherin. Alternatively, loss of lymph flow may be sufficient to inhibit VE-cadherin signaling without reducing its expression. Another related finding is that high levels of LTB_4_ in CRL negatively regulate the expression of VEGFR3 [15], which is part of the VE-cadherin mechanotransduction complex [100,101], and provides another way for inflammation to dampen mechanotransduction signaling required for valve maintenance. Therefore, future studies should investigate whether Th2 cytokines can negatively regulate VE-cadherin expression or cellular localization in the context of lymphedema or whether lymphedema affects mechanotransduction signaling and valve maintenance.

Finally, the order of events that lead to CRL need to be determined (Figure 1). Does inflammation cause a loss of lymphatic valves or instead respond to valve loss? Does loss of lymph flow directly cause valve regression or directly cause inflammation? Are lymphatic valve defects accompanied by increased lymphatic permeability and lymph leakage? The exact trigger that instigates the inflammation should be identified in future studies.

## Figures and Tables

**Table 1:** Genes mutated in congenital lymphedema that regulate lymphatic valve development.

Gene	Human Disease	Phenotype in Humans	Phenotype in Mice	Role in Valve Development
*FOXC2*	Lymphedema distichiasis (autosomal dominant)	Limb lymphedema, distichiasis, lymphatic vessel hyperplasia, abnormal pericyte recruitment [47].	*Foxc2*^−/−^ embryos are edematous and lethal, their mesenteries lack valves at E17.5 and they exhibit dermal lymphatic hyperplasia and abnormal pericyte recruitment [48,49]. Postnatal *Foxc2* deletion leads to valve regression and chylous effusion [36].	FOXC2 is a transcription factor that specifies lymphatic valve identity and regulates valve development during embryogenesis as well as valve maintenance postnatally.
*GATA2*	Emberger Syndrome (autosomal dominant)	Lower limb lymphedema and increased risk for developing myelodysplastic syndrome/acute myeloid leukemia (MDS/AML) [51,52].	Lymphatic-specific deletion of *Gata2* (*Gata^ΔLEC^*) in embryos led to edema, blood-filled lymphatics and abnormal pericyte recruitment. *Gata^ΔLEC^* exhibited significantly fewer valve specification clusters at E16.5 and mature valves at E18.5 in the mesentery. Postnatal lymphatic-specific deletion of *Gata2* led to regression of existing valves [34].	GATA2 is a transcription factor that regulates expression of genes required for valve development such as *Prox1*, *Foxc2*, *Nfatc1*, *Itga9* and *Fat4*.
*PIEZO1*	Generalized lymphatic dysplasia (autosomal recessive)	Non-immune hydrops fetalis which may or may not lead to in-utero demise, peripheral lymphedema, mild facial edema, chylous effusion [64,65].	Lymphatic-specific *Piezo1* knockout mice (*Lyve1Cre*; *Piezo1^cKO^*) exhibited pleural effusion and died shortly after birth. Their mesenteries revealed significantly fewer mature valves at P4. Lymphatic-specific deletion of *Piezo1* in adult mice led to regression of existing valves in the skin and mesentery [66,67].	PIEZO1 is a mechanosensitive ion channel that facilitates valve leaflet protrusion by stimulating LEC migration, actin polymerization and cell junction remodeling in response to shear stress signals.
*ITGA9*	Fetal chylothorax (autosomal recessive)	Bilateral chylothorax, hydrops fetalis [69].	*Itga9*^−/−^ mice develop chylothorax and die shortly after birth due to respiratory failure. Examination of their mesenteries at P5 reveal numerous incomplete valves with abnormal leaflets [68,70].	Integrin-α9 is a receptor on LECs that binds Fibronectin-EIIIA ligands in the ECM. This interaction facilitates FN matrix assembly outside the developing valve which promotes elongation of the valve leaflets.
*FAT4*	Hennekam syndrome (autosomal recessive)	Facial lymphedema, lymphangiectasia, mental retardation [57].	*Fat4*^−/−^ embryos exhibit subcutaneous edema at E14.5 and altered LEC polarity within developing vessels and they have significantly fewer mature valves in the mesentery at E18.5 [62,63].	FAT4 regulates LEC polarity in response to lymph flow which facilitates LEC alignment and migration during valve leaflet formation.
*RASA1*	Capillary malformation- arteriovenous malformation (CM-AVM) (autosomal dominant)	Vascular malformations in the skin and underlying tissues, limb lymphedema, chylous ascites, chylothorax, lymphatic hyperplasia [72,73,74].	*Rasa1*-deficient mice exhibit chylothorax, chylous ascites, lymphatic vessel dilation and hyperplasia, lack of smooth muscle coverage and valve dysfunction due to valve leaflet degeneration [76].	RASA1 is a RasGTPase-activating protein that negatively regulates LEC proliferation by suppressing Ras signaling. It also maintains functional valves by preventing LEC apoptosis.
*GJA1* (*Cx43*)	Oculodentodigital syndrome (autosomal dominant)	Digital anomalies like syndactyly, hypoplastic alae nasi, microdontia, ptosis, limb lymphedema [78].	Mice in which Cx43 is deleted in a lymphatic-specific manner have a high chance of dying from chylothorax at adulthood. They experience a delay in valve initiation and adult mutants have significantly fewer mature valves compared to WT controls [79,80].	Cx43 is a gap junction protein that is highly expressed in LECs on the upstream, luminal side of the valve and is required for valve leaflet formation. It colocalizes with Cx47 and maintains its levels in LECs of thoracic duct valves.
*EPHB4*	Nonimmune hydrops fetalis (autosomal dominant or sporadic)	Non-immune hydrops fetalis which may or may not lead to in-utero demise, peripheral lymphedema, atrial septal defect, varicose veins [81].	Lymphatic-specific deletion of *Ephb4* during embryonic development led to subcutaneous edema, blood-filled dermal lymphatics and lack of lymphovenous valve formation. Embryonic and postnatal deletion of *Ephb4* leads to a complete absence of lymphatic valves in the mesentery [81,82].	EPHB4 is a receptor tyrosine kinase that regulates valve development and maintenance through activation of its kinase domain upon binding its ligand EphrinB2.
